# Supplementary figures and images for: Statistical Analysis of Absenteeism in a University Hospital Center between 2007 and 2019
Source: Int J Environ Res Public Health. 2022 Oct 10;19(19):12966. doi: 10.3390/ijerph191912966 (PMC9565198; doi:10.3390/ijerph191912966)

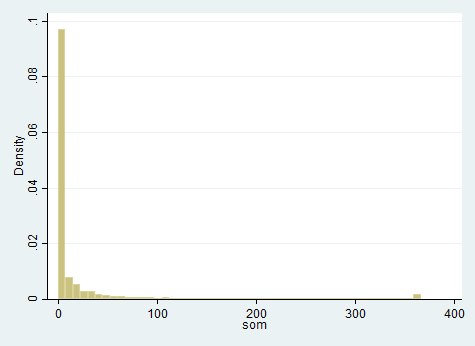

Supplement: Supplementary file 1 [file ijerph-19-12966-s001.zip › Supplementary Figure S1.png]

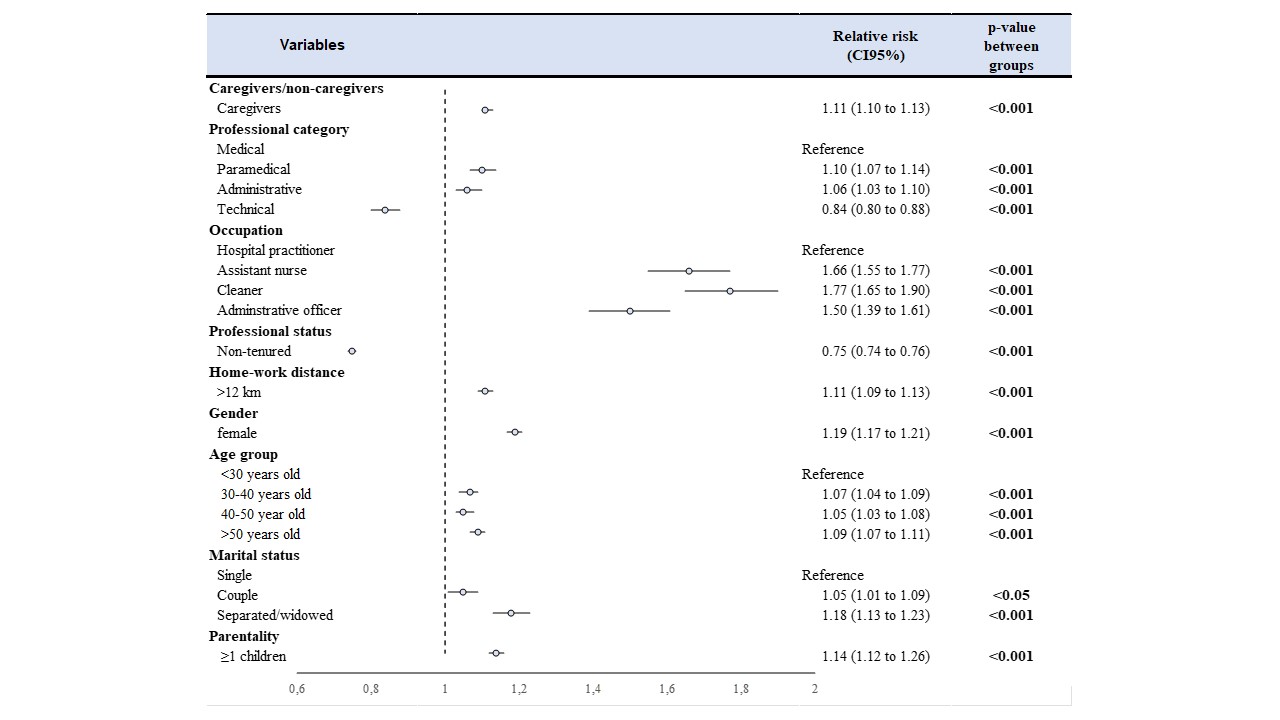

Supplement: Supplementary file 1 [file ijerph-19-12966-s001.zip › Supplementary Figure S2.jpg]

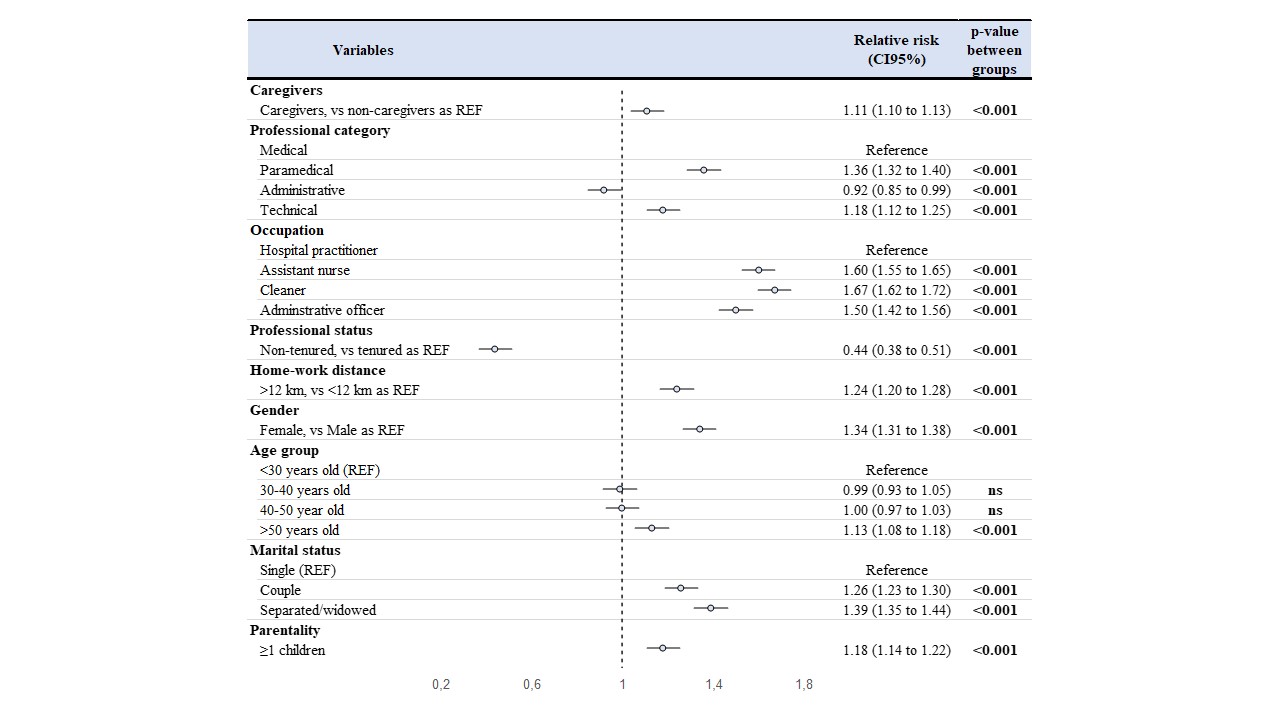

Supplement: Supplementary file 1 [file ijerph-19-12966-s001.zip › Supplementary Figure S3.jpg]

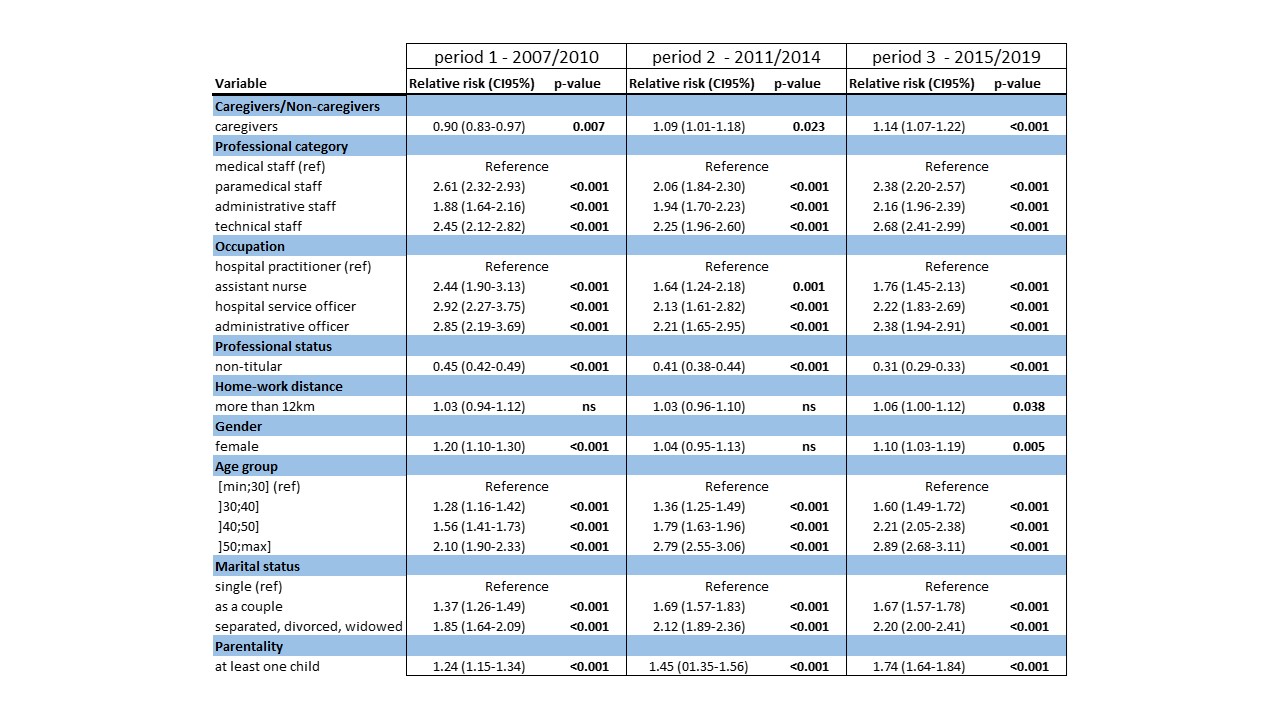

Supplement: Supplementary file 1 [file ijerph-19-12966-s001.zip › Supplementary Table S1.jpg]

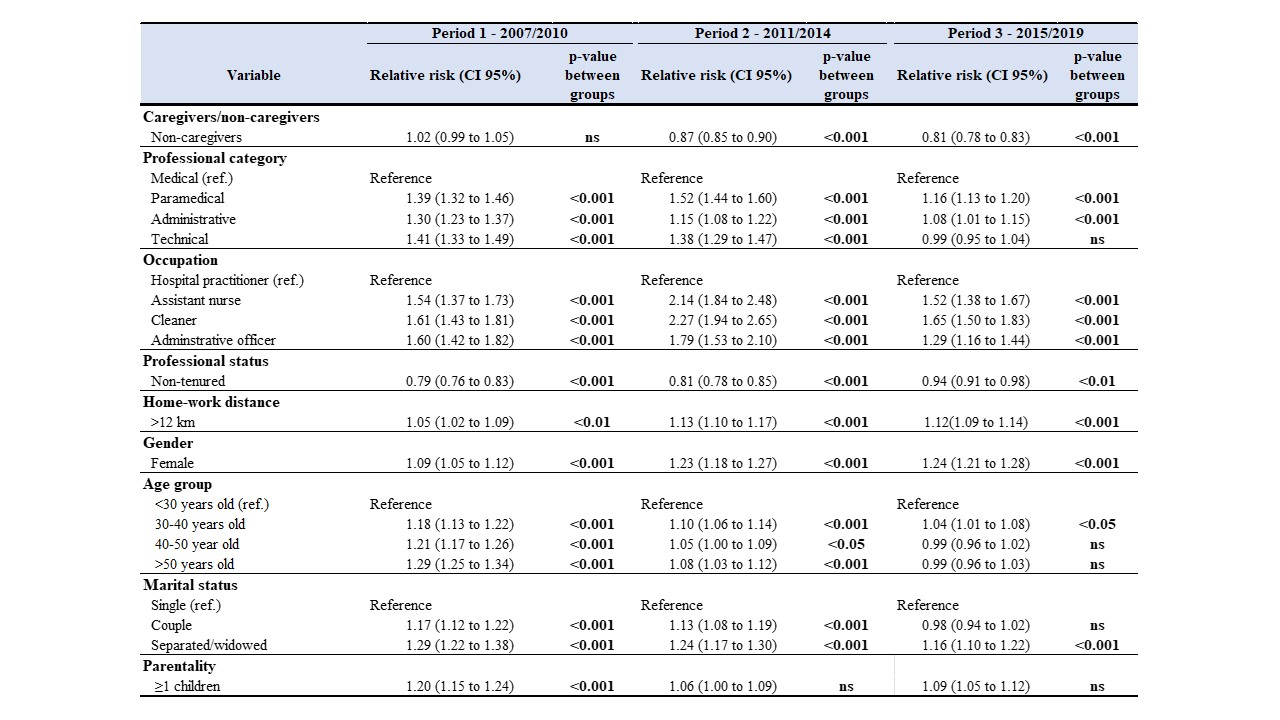

Supplement: Supplementary file 1 [file ijerph-19-12966-s001.zip › Supplementary Table S2.jpg]
